# Living and dying with incurable cancer: a qualitative study on older patients’ life values and healthcare professionals’ responsivity

**DOI:** 10.1186/s12904-020-00618-w

**Published:** 2020-07-20

**Authors:** Jelle L. P. van Gurp, Anne Ebenau, Simone van der Burg, Jeroen Hasselaar

**Affiliations:** 1 Department of IQ healthcare, Ethics of healthcare, Pain and Palliative Medicine, Radboud University Nijmegen Medical Center, P.O. Box 9101, 6500 HB Nijmegen, The Netherlands; 2 Department of Anesthesiology, Pain and Palliative Medicine, Radboud University Nijmegen Medical Center, Nijmegen, Netherlands; 3 Wageningen Economic Research, Wageningen University & Research, Wageningen, Netherlands

**Keywords:** Advanced cancer, Older persons, Palliative care, Life values, Patient outlooks on life, Patient perspective

## Abstract

**Background:**

In ageing Western societies, many older persons live with and die from cancer. Despite that present-day healthcare aims to be patient-centered, scientific literature has little knowledge to offer about how cancer and its treatment impact older persons’ various outlooks on life and underlying life values. Therefore, the aims of this paper are to: 1) describe outlooks on life and life values of older people (≥ 70) living with incurable cancer; 2) elicit how healthcare professionals react and respond to these.

**Methods:**

Semi-structured qualitative interviews with 12 older persons with advanced cancer and two group interviews with healthcare professionals were held and followed by an analysis with a grounded theory approach.

**Results:**

Several themes and subthemes emerged from the patient interview study: a) handling incurable cancer (the anticipatory outlook on “a reduced life”, hope and, coping with an unpredictable disease) b) being supported by others (“being there”, leaving a legacy, and having reliable healthcare professionals) and; c) making end-of-life choices (anticipatory fears, and place of death). The group interviews explained how healthcare professionals respond to the abovementioned themes in palliative care practice. Some barriers for (open) communication were expressed too by the latter, e.g., lack of continuity of care and advance care planning, and patients’ humble attitudes.

**Conclusions:**

Older adults living with incurable cancer showed particular outlooks on life and life values regarding advanced cancer and the accompanying last phase of life. This paper could support healthcare professionals and patients in jointly exploring and formulating these outlooks and values in the light of treatment plans.

## Background

One of the recent foci of healthcare systems in Western countries has been the treatment for and care of a growing number of seriously ill older persons. Due to aging populations, the prevalence of chronic diseases has been rising. This includes an increase in the incidence of cancer [1]. Older persons with cancer have become one of the dominant patient groups for healthcare professionals (HCPs). However, little is known about how cancer treatment trajectories and/or supportive care affect the outlooks on life and underlying life values of often multimorbid and functionally declined older patients. Such trajectories may cause a shift in their outlooks on life and life values [2]. We consider life values as “desirable states, objects, goals or behaviors that a person uses to reflect on and evaluate personal actions and life events, as well as the actions and lives of others. Life values transcend specific situations” [3] and are embedded in a person’s outlook on life or foundational reality [4].

This article focuses on how diverse outlooks on life and life values of older adults living with incurable cancer shape their experience of the palliative care phase. HCPs are expected to know and communicate with patients about such issues [5–7]. Creating open and understandable communication about life values requires sincere attention to patient stories, which are often interwoven into major illness-related problems [8, 9]. In practice, though, mutual understanding of outlooks and life values between professionals and patients is not established easily due to (lack of) specifically required communication skills, e.g., a listening attitude and knowing age-specific language [10]. Furthermore, in-depth communication might be hindered by hospitals being cure-oriented. Such cultures might not provide much room and language for beyond-cure scenarios, i.e. palliative care [11].

Furthermore, older patients themselves report experiencing difficulties in expressing their outlooks on life and accompanying life values. Communication about these often implicit issues can be complex as it requires self-reflection, analytic competence, and articulation [12]. Research shows that, although they desire to be respected as persons, older person s’ abilities to be expressive about their personal desires is complicated by loss of self-identity, self-esteem and certainty about the future and their complex health needs and situation [3, 10, 13–18]. Furthermore, some of these older patients tend to avoid ‘bothering’ others, their own family members included [17, 19] and to leave important decisions to doctors in whom they have and put great trust [13, 20, 21]. Some studies show that older people, indeed, are more physician-oriented and do not seem to prefer active roles in decision-making but passive roles and trusting and person-centered relationships with their doctor instead [22–25]. However, other research suggests that older patients seem to desire shared or collaborative decision-making just as much as younger patients and that it positively influences patients’ satisfaction with their physicians [26–28]. With regard to this heterogeneity of older patients’ preferences for involvement in decision-making and for communication, different approaches to person-centered care may be needed. An exploration of life values is key to such approach [18, 25, 29].

This article will include both the views of patients about their life values in perspective of their treatment and the views of professional caregivers with regard to (including) these values. It aims to describe: a) outlooks on life, including the values that are central to these outlooks of Dutch older adults living with incurable cancer and; b) how various palliative care professionals customize their treatment and care to these outlooks and values.

## Methods

### Study design and rationale

The objective of this research project was to develop a digital value-clarification tool for older persons living with incurable cancer. Such tools can support patients with reflecting on personal outlooks and underlying values, with articulating their perspectives related to care and treatment needs, and with “producing better congruence between values and choices” [30]. Provided that value-clarification tools are sensitive to a personal logic, based on the patients’ emotions and contexts, as well as integrate patient-reported preferences with the appropriate information [30, 31], these tools are thought to be helpful for the participation in the shared decision-making processes that are desired by older persons with cancer [26, 27].

In preparation for the development of a value-clarification tool it was considered important not only to investigate the outlooks and life values of patients, but also to get an impression of the ways in which palliative care professionals, including oncologists, would respond to patients expressing these outlooks and values in daily care. Therefore, in 2014, a qualitative study was carried out that included single semi-structured interviews with 12 older persons (≥ 70 years of age) with incurable cancer to discover their life values and outlooks on life, and two single group interviews with healthcare professionals (*n* = 10) involved in the care for these patients.

### Sampling and inclusion

For the sampling of older persons, convenience sampling [32] was performed, but with as much focus as possible on guaranteeing variety in (older) age groups, gender, place of residence, and marital status. Participants were recruited from a geriatric oncology ward of the Radboud University Medical Center, primary care clinics, a nursing home, and via homecare institutions, which are all situated in or around the city of Nijmegen, the Netherlands. Initially, all participants were approached face-to-face by a professional caregiver close to them. Participants were eligible if they were: a) aged 70 or older; b) aware of having incurable cancer; c) able to speak Dutch and; d) not cognitively impaired. A professional involved in the care for a patient was asked to assess the latter. JG contacted them afterwards by telephone. Drop-out and exclusion were not counted.

With regard to the group interviews with HCPs, a variety of professionals who had experience in working with older adults living with incurable cancer and who were based in the large regional healthcare network around Nijmegen were selected and directly invited by means of e-mail to participate.

### Data collection and setting

First author JG (male, experienced qualitative researcher; social scientist/ethicist) conducted the individual, semi-structured interviews with the older persons with cancer at their preferred place (11 at patients’ homes and one at a hospice). For the development of the interview guide, a narrative approach was followed. In this approach patient stories are “a way of redrawing maps and finding new destinations.” [33] In comparing the life before and after falling ill, telling what the old destinations were and what the new destinations have to be, participants will implicitly talk about what they consider important in life. The interview guide (Additional file 1) starts with the experience of being ill and being related to a social environment, followed by asking for stories about how respondents interact with their social environment. The third section is explicitly about the new goals and destinations in a life with illness, and the fourth section about their own role in realizing these goals. In this fourth section there is also attention for the comparison between destinations in life prior to being ill and the destinations in life when being ill. Patient interview guides were peer reviewed by the project group, i.e. authors, and an independent GP, experienced with this patient group and interviewing. During three interviews, a patient’s proxy joined who, however, did not intervene with the interview.

The group interviews with HCPs were held at two neutral locations (hospitals). A semi-structured topic list (Additional file 2) was developed including seven typical case descriptions based on the results from the patient interviews, which functioned as vignettes. The HCPs were asked for: 1) recognition of the values expressed by the patient participants; 2) reactions to such value expressions; 3) responsibilities and preferences in including value expressions in decision making and caring for this particular patient group and; 4) barriers and facilitators in providing patient-tailored care and patient-centered communication. An independent facilitator moderated the group interviews. JG and AE were present at the group interviews. The HCP interview guide was peer reviewed by JG, AE, SB and the independent facilitator.

All interviews were audio-recorded, and transcribed verbatim by the second author, AE (female, experienced qualitative researcher; anthropologist). Fieldnotes were made during and after interviews, which provided the interview with the necessary context to start the analysis. Neither patients nor HCPs received the interview transcripts. No prior relationship existed between the interviewers and the patient participants. The researcher knew some HCPs only from previous research-related activities.

### Analysis

The data of the patient interview study were analyzed using open and axial coding, common to a grounded theory approach [34, 35]. After uploading the data to The Qualitative Data Analysis and Research Software ATLAS.ti version 7.0, two data coders (JG and AE) started to apply open coding to the first four interviews. All codes and accompanying memos were discussed and adapted until agreement was reached and a list of initial codes was formulated [36, 37]. Both coders then independently categorized the initial codes of the four interviews, applying a “constant comparative analysis” [34]. In the next step, joint axial coding was used to see if these provisional categorizations could be linked into coherent schemes, and to label and rank these categorizations by ascribing themes and subthemes representing the experiences important to the participants. In addition, memos were written on the relationships between (sub)themes and various outlooks on life and life values [38].

With this initial scheme containing themes and subthemes, JG and AE coded two similar interviews and checked again for agreement. In this step, data saturation was reached; no new codes and themes came up [36, 37, 39]. Then, the improved scheme was used by JG and AE to independently analyze the remaining six interviews. Their continuous peer debriefings ensured further refinement of the scheme and, thereby, the credibility of the emerged themes and subthemes. In addition, the scheme was subjected to multiple in-depth peer reviews in the collaborative research group. The final step of the analysis was an explication by JG and AE of the values that were at stake in each theme or subtheme. This additional step was necessary as such values usually remained implicit in communication about experiences and in the patient interviews as well.

The data of the group interviews were analyzed separately, however, by one data coder only (JG). He followed the abovementioned procedure. Although the results of the two group interviews were similar, data saturation on some aspects was not reached. As the fictitious case descriptions used for the group interviews were based on the results of the interview study with patients, the group interviews offered an opportunity to subject the results from the interview study with patients to in-depth reviews from professional caregivers. These were not meant as a formal member check though.

## Results

### Characteristics of patient participants and HCPs

Twelve older (> 70 years) patients with incurable cancer participated in the interview study. Nine males and three females were included. Their age ranged from 73 to 91 (Table 1). Interviews ranged from 52 to 84 min. The two group interviews took 116 and 118 min and were joined by 10 HCPs in total (two oncologists, two spiritual caregivers, four geriatric specialists, one family physician and one oncology nurse) who had extensive working experience with older adults living with incurable cancer.

**Table 1:** Patient participants’ characteristics

Participant number	Age (range, from - till)	Gender	Diagnosis
1.	85–90	M	Gastric intestinal cancer
2.	75–80	F	Endometrial cancer
3.	70–75	M	Pancreas carcinoma
4.	80–85	F	Breast cancer
5.	75–80	M	Prostate carcinoma
6.	80–85	M	Gastric intestinal cancer
7.	80–85	M	Lung cancer
8.	80–85	M	Multiple myeloma
9.	70–75	M	Carcinoma of the ampulla of Vater
10.	90–95	F	Breast cancer
11.	80–85	M	Renal cell carcinoma
12.	70–75	M	Prostate carcinoma

### Findings

From the interviews with older adults living with incurable cancer, three main themes and several sub-themes emerged: 1) handling incurable cancer (the anticipatory outlook on “a reduced life”, hope and coping with an unpredictable disease); 2) being supported by others (“being there”, leaving a legacy, and having reliable healthcare professionals) and; 3) making end-of-life choices (acceleration and alleviation of death, and the place of death). Below, each of these main themes will also be contrasted with and reflected upon by the data from the group interviews with healthcare professionals.

### Main theme 1: handling incurable cancer

#### Anticipatory outlook on “a reduced life”

In the interviews, anticipatory perspectives on the turning point when life becomes *“a reduced life”* and no longer worth living, had a prominent place. Such a life was described as largely meaningless and something that needed to be held off as long as possible. It is a life in which patients are no longer able to fully engage in their life-worlds. On several domains, life appears conditional. First, “a reduced life” was described by participants in physical terms as severe pain and an emaciated body, which both were said to devour a human being and affect human dignity (Table 2, Q1). The same would account for a lack of mobility (Q2a, b) Furthermore, this conditional life was defined in mental terms as losing one’s cognitive health and living with depression (Q3). Participants aimed at keeping a balance between negative side-effects of treatment and their life values (Q4).

**Table 2:** Patient participants’ interview quotes

**Main theme 1: Handling incurable cancer**		
**Sub-themes**	**Related life values**	**Representative quotes**
*1.1 Anticipatory outlook on “a reduced life”*	Being free of pain or pain being livable (autonomy)	**Q1:** “I think pain is terrible. You’re powerless when it comes to pain. […] It is awful to have such pain, and being unable to do something about it.” - i6 (male, 83 years old, widower, four children)
	Having energy; maintaining mobility; preserving control over bodily functions (autonomy, dignity)	**Q2a:** “… if you have to lie in bed constantly. That you won’t come out of it anymore. I consider that to be undignified.” - i9 (male, 74 years old, widower, no children)
		**Q2b:** “My world is shrinking as I am lying in my chair. Just watching, doing nothing.” - i8 (male, 83 years old, married, three children)
	Preserving mental and cognitive capacities (autonomy)	**Q3:** “Well look, I am so happy that my mind is still in order. If that would decline, I would be sitting there like some sort of zombie. I would find that awful.” - i10 (female, 91 years old, widow, no children)
	Proportionality; balance between treatment and quality of life (beneficence and non-maleficence)	**Q4**: “Well, after I quitted with chemo, apparently that is when you regain some energy. I could even do more with filming [hobby].” - i5 (male, 76 years old, widower, three children)
	Becoming attentive to small things in life (being present, being attentive and connected with the small things)	**Q5:** “I have a different perspective now. I can really enjoy the small things in life that I usually took for granted.” - i12 (male, 73 years old, in a relationship, three stepchildren)
*1.2 Hope*	Preserving an open future; combining hope with realism	**Q6:** “I do hope I can live a little longer, but you never know. I have cancer and that’s a fact.” - i3 (male, 74 years old, married, two children)
	To partake in “normal life” as long as possible (continuity)	**Q7:** “No, actually I don’t have any wishes left, only that I am allowed to live a few more years, that would be good. I don’t have to reach the age of ninety.” - i6
	Preserving a positive outlook on life (positivity)	**Q8:** “I hope, and it is not in my nature, that I will not become downhearted. […] This is how it is and I have to accept that. Look, if I were thirty years younger, then it would be a different story maybe. But from the diagnosis I think: this is how it is.” - i2 (female, 79 years old, married, two children)
*1.3 Coping with an unpredictable disease*	Surrender to the disease as part of the acceptation process (acceptation)	**Q9a:** “I do not work in the garden anymore. I have a gardener now, he’s working the garden. But I mean, you have to let go of those activities.” - i10
		**Q9b:** “With this disease that I have, it [death] can happen at any moment […] Right now, I’m living here. With the health I have left, because right now I’m feeling perfectly fine. […] We cannot determine the diseases’ endpoint.” - i8
	A good death is a death after a long and rich life	**Q10:** “And I have a whole life behind me. At this moment, I think I can leave it like that. […] However, as long as I am still hale and hearty, I enjoy life.” i10
	To finish your thoughts brings peace (closure, peace of mind)	**Q11:** “That was the most pressing thing back then. I was at a loss what to do: damn, if I had a breakdown nothing would have been arranged. And this is how I got with [organization]. The arrangements that we made gave me some room to breathe. Made me lead a more careful life. […] Everything is in order now.” - i11 (male, 83, divorced, lost contact with his children)
**Main theme 2: Being supported by others**		
**Sub-themes**	**Related life values**	**Representative quotes**
*2.1 “Being there”*	Receiving love from one’s fellow human beings: family, friends, religious communities (charity, support)	**Q12a:** “**What does it mean to you that they are there for you?** A lot. It means I have done well. I get a little pat on the back.” - i6
		**Q12b:** “I visit the church every Sunday. If I can. […]. I do it because I’ve always done it, you get used to it. You know everybody [laughs]. That provides real support. Well, and I also do my evening prayer every night. These old habits.” - i6
	Preserving the capacity to be open to your social environment (being connected with others)	**Q13:** “It was very hard to understand my new situation. To find my way back to my family. You have to be able to express your new situation; to find words that fit.” - i8
	To have discussions about the meaning of life (meaningfulness)	**Q14:** “[I talk] about everything. I talk with my friends and family like I am talking with you now.” - i2
	Maintaining independence	**Q15:** “I am trying to do it myself as much as I can. My daughters support me, if they are able to. Luckily, till now, all goes well. I hope this will last a while.” - i5
	Not to become a burden to others	**Q16:** “Well, then you never get out [your home]. I do not like the sound of that. On a few square meters forever […] And from my children I can’t expect that they will be with me 24–7.” - i12
*2.2 Leaving a legacy*	Sharing ideas/objects of value with others/ still contributing to society	**Q17a:** “Sure. We already took care of it all. The kids all know what I have saved and what they will receive. I have got no secrets.” - i6
		**Q17b:** “Currently I am restricted to the bedroom, dining room, and… Listen, if I still, for example, could help those young doctors with communication, I feel useful. I am useful within society […]. If I would have stayed within that tiny room at home, I would have been useless.” - i12
	Peace of having arranged all matters important, e.g., finances	**Q18:** “I already have an executor who takes care of all that needs to be done. Funeral. I already made my death notice. Yeah. I am like “I take care of that, I will write it myself”. They don’t have to [bother] the neighbors…” - i10
	Continue to support a vulnerable partner (responsibility)	**Q19:** “Right now, I’m thinking about what would happen to my demented wife when my health declines. I’m worried about these things.” - i2
*2.3 Having reliable healthcare professionals*	Being acknowledged as a person; the empathic professional (humanity)	**Q20a:** “Paying attention. That is, communication as well. And so, I told him [the physician]. I said you know much about medication and side-effects, but people have side effects too. When you hear something, everything inside explodes. However, you [physician] keep on talking. Instead, you could have waited a bit or asked me whether I understand what is told.” - i12
		**Q20b:And what about that nurse makes him so nice?** Well, he is very kind, he receives you very kindly. And then, he says, “such cold hands you have!” [laughs] [..] I like that. […] They are interested. And if they visit me, they say “good morning, how are you?” - i10
	Continuity in care and support	**Q21**: “I had a different doctor every time and so, we wrote a letter and stated that we did not appreciate this […]. Then, we got doctor [name] and he is there as much as possible.” - i2
	Being provided with honest information (honesty)	**Q22:** “I find him very professional. Open, he tells and shows everything.” - i5
**Main theme 3:*Making end-of-life choices***		
**Sub-themes**	**Related life values**	**Representative quotes**
*3.1 Acceleration and alleviation of death*	Maintaining autonomy and dignity, controlling your own life	**Q23a**: “I am not against termination of life. I would rather end life consciously than slowly degenerating. I have to leave before it muddles along […] If I would let myself die [naturally], I would face a period with death-struggle and misery. Getting old and dying without misery, that is euthanasia […] If I would get really ill and need assistance… if I lose that independency, I want to get out. Then, my time has gone.” - i11
		**Q23b**: “I don’t want to, I have it here, be reanimated. I’ve asked for a medallion, because when your brain doesn’t function for 6 min, your mind is gone. Then, it is only the nursing home where you can sit all the time. I don’t want that.” - i10
*3.2 Place of death*	Being at home; carrying a personal history; preserving authenticity	**Q24:** “**What is the value of being home at that moment [of dying]?** Well, we built that [home] together. I value that. We worked hard on it, together.” - i5

With patient participants being used to a slow decay that comes with an older age, the pace of current deterioration accompanying advanced cancer frightened many participants. Another complicating factor for older people in handling their incurable cancer was multimorbidity. Participants, for example, also suffered from heart problems, from an upset stomach as a consequence of previous radiation, or problems originating from his working life with dust affecting his organ of balance and ability to sleep. Most participants reported living with multiple diseases, which usually hindered intensive cancer treatment and increased the burden of care for proxies. However, instead of focusing on future fears and further decline, some patients also described that being confronted with life-threatening disease, made room for more attentiveness towards the small things of life in the present (Q5). Also, participants admitted that their anticipatory outlooks on “a reduced life” might be dynamic as they had been flexible and resilient during earlier episodes of suffering.

#### Hope

Also central to participants’ interviews were different sorts of hope, e.g. the hope for extension of life, including receiving life-extending medical treatment. Although hope for a miraculous cure was sometimes expressed, treatment was mainly expected to support a calm continuation of life. Patients stated to be realistic (Q6). Patients talked about a life that is dedicated to partaking instead of creating. They strongly desired to maintain their “ordinary life” (Q7). Finally, participants spoke of a desire to maintain a positive attitude (Q8), although they also stated that worrying is necessary to deal with disease-related thoughts.

#### Coping with an unpredictable disease

The stories of hope reflect the value of having a future. However, this future is constantly challenged by the unpredictability of cancer. Contributing to the unpredictability is cancer’s appearance as a largely “hidden visitor”, only recognizable through medical examinations and imaging. To cope with the insecurity that accompanies advanced cancer, participants need resilience; they reported on continuous adaptation processes in response to slowly losing parts of life to illness as well as a surrender to this process (Q9a, b). Resilience appeared to be a balancing act between being resigned to the outcome of the illness and actively seeking new treatment opportunities. Also, there appeared some friction between acceptation, usually formulated as dying with the knowledge of having lived a “long and happy life” (Q10), and subtle forms of denial: participants talked about postponing thoughts about severe illness and death while trying to maintain an “ordinary life” and/or blamed their physical decline on age. Others put their mind at rest by making future-arrangements with regard to their health (Q11). Table 3 shows how HCPs stated to respond to the sub-themes of ‘Handling incurable cancer’.

**Table 3:** HCPs’ reflections on main theme 1: Handling incurable cancer

Subtheme	Clinical practice
*1.1 Anticipatory outlook on* “*a reduced life”*	The HCPs participating in the group interviews recognized the desire of older patients to continue normal life as long as possible. To respect this desire or “protect” patients, HCPs could propose not to continue with chemotherapy. In such cases, “supportive care” aiming at symptom control could be explained and presented as being the best option. Whether patients accept this focus on supportive care, largely depends on how they think curative treatment is going to contribute to better quality of life now and in the future. The material of the group interviews, furthermore, showed that HCPs attach great importance to older patients’ social systems, with special attention to the relationships between parents and children. These long-standing relationships usually influence the balancing act between “normal life” and treatment through implicit and explicit expectations that shape care-giving and care asking. For example, HCPs experienced that they were often awarded the power of decision-making by their older patients so that they could protect them from their more cure-oriented children.
*1.2 Hope*	According to HCPs, patients usually hope for action by HCPs. The latter thus have a responsibility to explore a patient’s hope. Another goal for HCPs, as they stated, is to keep the hopes of patients realistic, incorporating information, scenarios, and “the insecurity of not knowing exactly” into their conversations with patients. Realism entails understanding that the body is quickly deteriorating while the mind can still be sharp. If this is the case, HCPs stated that palliative care should be presented as the appropriate action. This might, at first sight, diverge with patients focusing on life extension primarily.
*1.3 Coping with an unpredictable disease*	HCPs discussed that cancer might be experienced by patients as an abstract, unobservable phenomenon that requires optimal medical imaging combined with HCPs’ advanced communicative skills to be at all understandable. HCPs feel they have a responsibility in this matter, in line with the preferences of their patients.

### Main theme 2: being supported by others

#### “Being there”

Participants usually started the second part of the interview with naming practical assistance by family and friends, e.g., in the household. However, the essential value of these proxies was hidden in the expression of “being there” to distract from worrying thoughts, to be there despite a patient’s physical and/or mood changes, and to be there until the very end. The support was considered meaningful (Table 2, Q12a, b). Proxies being there, though, required some effort on being open to loved ones on the patients’ side too as the occurrence of cancer could completely disrupt social relationships (Q13). For some patients, the realization of their own vulnerability and mortality intensified relationship with their proxies, which included intersubjective searches for the meaning of life (Q14). Others, however, enjoyed some attention for their well-being and sorrow, but did not want their disease story to be dominant and asked their proxies to support them in living the “ordinary life”, preferably with a slight touch of humor. Furthermore, participants expressed a desire to maintain their independence as long as possible (Q15) as well as a reluctance to burden others (Q16).

#### Leaving a legacy

In light of an approaching death, some participants reflected on the value of their life for others and on leaving some kind of legacy, e.g., wisdom, valuable personal objects, inheritance for proxies (Q17a) or society (Q17b). For some participants practically arranging their legacy provided peace (Q18). However, for others daily life did not offer room for personal reflection on life, illness, and death as they had to take care of an equally ill partner that demanded much attention (Q19). This finding alludes to a life that cannot be focused on partaking and reflection but is instead about creating the optimal life for one’s partner.

#### Having reliable healthcare professionals

In the interviews, participants discussed valuable interactions with HCPs. First, the insecurity of having cancer requires personal attention of HCPs who reserve time for their patients’ stories, listen closely and are attentive and outreaching (Q20a, b). Second, a continuous and select group of HCPs who are reachable and available, furthermore, is appreciated, however, not always experienced as such (Q21). Finally, participants stated to prefer honest, straightforward, and complete information (preferably supported by numbers or images with regard to the invisibility of cancer) but expressed some ambivalence as information should be provided gently, i.e. attuned to a patients’ hopes (Q22).

In Table 4, HCPs’ reactions to the sub-themes of ‘Being supported by others’ are explained.

**Table 4:** HCPs’ reflections on the main theme 2: Being supported by others

Subtheme	Clinical practice
*2.1 “Being there”*	HCPs recognized patients’ reluctance to burden their proxies. Some HCPs stated to try to discover what patients mean by burdening; did proxies indeed state not to be willing to be burdened or is this a patient’s pre-assumption?
*2.2 Leaving a legacy*	Some HCPs experienced that patients indeed reflected upon their lives, however, only if patients were still “clear-headed”. Other HCPs stated chaplains, mostly, were involved in such existential issues. In this light, the importance of multidimensional care was expressed. Furthermore, in the group interviews, HCPs considered the fact that older persons increasingly have to care for one another is one of the important issues for future healthcare and that by this, patients’ own coping processes could be neglected. This was confirmed in some patient interviews. Instead of immediately trying to solve this and arrange things, professionals also do well by getting to know the patient together with his/her social system, the responsibilities patients bear for partners and/or family and potential fears over losing control over their situation. Creating room and some peace of mind could lead to patients acknowledging their personal dying process. HCPs also mentioned the involvement of a case manager specialized in dementia. Such a professional has a role in anticipating on future scenarios and in unburdening the older person. Nowadays, HCPs stated, recognizing the patient’s needs, often, comes too late.
*2.3 Reliable healthcare professionals*	HCPs acknowledged that an explorative dialogue with (older) patients is essential to learn about their concerns. However, they experience some barriers in talking to older patients: in general, they consider older people less assertive, more modest and more willing to hand over responsibility to HCPs. HCPs stated that shared decision-making “is not for everyone”. In such cases, HCPs try to trust their own gut feeling about what is good for the patient. An additional issue is patients’ lack of time for reflection due to the usually quick succession of news, scans, and treatments. Furthermore, HCPs agreed with patients’ desire to have a central and available HCP instead of changing HCPs. Continuity could stimulate a trusting relationship between patient and HCP.

### Main theme 3: making end-of-life choices

#### Acceleration & alleviation of death

The anticipatory fear of pain and/or an emaciated body was the most prominent reason for thinking about how to direct and control one’s end-of-life and death. Most participants aimed to prevent the occurrence of long-term suffering, which was considered meaningless. Often, patients’ fears were a result of experiences with cancer within their social network. Participants pondered on advance directives, which were mostly focused on ‘Do Not Resuscitate orders’ or euthanasia requests, although they were unsure if and when to put these directives into action (Table 2, Q23a, b). This subtheme overlaps with theme ‘1.1 Anticipatory outlook on “a reduced life” ‘.

#### Place of death

Home was the preferred place of death for the patient participants, but for patients with little social connections, an impending neglect and/or a solitary death made preferences shift towards specialized healthcare facilities, e.g., a hospice (Q24). Table 5 discusses how HCPs relate to the sub-themes of ‘Making end-of-life choices’.

**Table 5:** HCPs’ reflections on main theme 3: Making end-of-life choices

Subtheme	Clinical practice
*3.1 Acceleration & alleviation of death*	HCPs mentioned that patients’ request on acceleration and alleviation of death was a consequence of patients’ fears of “the big unknown”. However, they experienced that older patients’ ideas about the end-of-life are usually more veiled as the patient interviews also suggested, or too simplistic; for example, patients talk about “being put to sleep.” With respect to euthanasia requests, HCPs notice a lack of knowledge about its proper realization. HCPs noted that a shared exploration of a patient’s fears, previous experiences with cancer, motives, expectations of the disease trajectory and social situation, and potential scenarios, including special forms of treatment, such as palliative sedation, should at least precede its actualization. Euthanasia and other end-of-life decisions ideally are slow decision-processes in which proxies and healthcare professionals have an opportunity to accompany the patient and prepare for it themselves. Proactively providing clear information about future scenarios, alternatives and end-of-life requests is important in such a process. HCPs, though, stated advance care planning is not a common practice yet.
*3.2 Place of death*	To provide the best patient-centered care, some HCPs believe that a diverse but select group of HCPs should be involved in care. Also, the informational “gap” between the hospital and general practitioner (GP) should be decreased. Sometimes a GP is not informed about a patient situation and neither in touch with a patient as this latter is still in hospital treatment. However, according to HCPs, patients tend to ask questions to their caregivers related to his/her specialism, e.g. asking an oncologist for cure. A GP, however, could have a “coaching” and preparatory role in making decisions related to, e.g. a home-situation and dying at home, and thus, should be involved in care.

## Discussion

From the data, three main themes emerged: handling incurable cancer, being supported by others, and end-of-life decisions; each domain contains several outlooks on life as well as the underlying values of older adults living with incurable cancer. The data from the group interviews show how HCPs recognized and responded to such outlooks and values.

### The older person living with incurable cancer

Anticipatory fears of loss of, e.g. the body, the mind, and social contacts play an important role in the lives of every patient, including older adults living with incurable cancer [40]. This study shows that these fears are usually influenced by previous experiences with cancer in their social network. HCPs acknowledge that these fears should be explored, but exploration is not a simple task as patients can be reluctant or yet unable to share these often-slumbering fears. The literature suggests that the origin and impact of these fears can be explored through guided dialogues with patients, in which they might find their way back out of their burdensome thoughts [41]. Although healthcare professionals are willing to aim for such dialogue, they expressed some barriers, e.g., older people being less assertive and less in need of being in charge. This is also reported in a literature review about life values of older adults living with incurable cancer [3], however, not expressed by patient participants in our study. Other barriers expressed were patients and HCPs both experiencing lack of time for reflection and slow decision-making processes, and cancer being an intangible phenomenon.

This study describes the kind of lives older adults living with incurable cancer hope to live and keep on living for as long as possible. It appears to be a life more focused on partaking instead of creating [42]. The latter belongs to previous life phases, whereas the combination of older age and illness appears to motivate a willingness to be absorbed into present-day life. Congruently, the literature shows patients’ desire for continuity and conservation of the activities they used to do and the person they have always been [3]. Both patient participants and HCPs indirectly stressed the importance of a careful use of the prejudice that dying at an old age is an inherently good death taken in consideration that one has lived a long and meaningful life [43]. Nevertheless, older patients seem just as much focused on the continuation of life as younger patients might be. A precondition to the wish to continue life seems to be that this future life still predicts some quality of life: talking from a position of relative good quality of life, even our oldest participant admits that she wishes to continue her life provided that it brings her the opportunity ‘to enjoy the things in life’. Although this seems to apply for all participants, the limited sampling in certain aging groups limits this conclusion when it comes to the oldest old (*n* = 1 in this study).

### The importance of the social context

A patient’s social context should be seen as the result of years of often-dynamic family and friendship constellations. Children play an important role in their parents’ treatment decisions and often have a role in informal care and information provision [44, 45]. On the one hand, some patient participants showed that they tend to relate this informal support to the answer of two questions, namely, ‘Have I done as I should have?’ and ‘Have I been a good parent?’. On the other hand, it was shown by patients and HCPs from this study as well as the literature, that older adults living with incurable cancer try not burden their family members too much [3, 46]. Furthermore, HCPs emphasized the need for attention to the patient’s social system in light of the, sometimes, more cure-oriented children. Palliative care, preferably timely and multidimensional, should be explored together, i.e., by HCPs, patients and proxies.

### On a societal level

With increasing ageing [1] and - at least in the Netherlands - a change from classical welfare states to participation societies (systems built on, among others, citizen participation, family care, and self-reliance) [47], it is to be expected that the care for older people increasingly comes from older people. Although the focus on a seriously ill proxy could distract from one’s personal ailment, it seems that the patient participants generally would have preferred to have some time and room to reflect with others on their disease, personal sorrow, and approaching death, round off the last phase of life, and achieve a good death. This article, however, provides a preview of a possible future society in which time for such reflection and closure seems at stake.

### Practical implications

This study emphasizes that HCPs could be more open to exploring older patients’ outlooks on life and life values and could pay more attention to the relationship between personal values and family and social positions. In this study’s interviews, participants constructed a moral landscape: a comprehensive story with which one could reflect on life, self, other persons, and former actions [48–50]. Moreover, they exchanged a vocabulary with which they could together set out to find new and workable imaginations of the patient’s future life. The interviews demanded considerable time, as the participants needed time to find the right words to reflect on their personal situation. HCPs recognizing this, however, stated to still have trouble organizing such shared explorations. To support older adults living with incurable cancer in considering and formulating their outlook on life and additional values before or after their visit to HCPs to efficiently and thoroughly prepare for shared explorations and decision-making processes. The themes and life values that arose from this research will be incorporated in a digital value clarification tool (in the form of a serious game; older adults who have advanced cancer can use this ‘game’ to learn about their values and preferences in ‘playful’ interaction with the game’s avatars. With the knowledge they gain from this game, older adults are expected to be better equipped to discuss their situation with their oncologists) [30]. In line with the MORECare statement, there is a theoretical model underlying this value clarification tool [elaborated on in a different paper; under construction], and the tool will also be thoroughly tested for feasibility and assessed for outcomes. Such a value clarification tool is not thought of as a replacement, but as a supportive tool for patient-professional dialogue. It is supposed to help patients formulate their most fundamental thoughts concerning their outlooks on life in an encounter with health care professionals, on the condition that the latter are open to discussing these matters [51, 52].

### Strengths and limitations

This article has a particular focus on seriously ill older persons, explaining the connection between the experiences of older adults living with incurable cancer and the reactions of the involved HCPs. It provides insight into the lives of these older persons as well as suggestions for appropriate communication and care. Although the data collection and analysis was thorough, this research also has its limitations. The most prominent limitation is a restricted time frame that made a cyclical process of collection, analysis, member check and collection etc. hard to realize. We lacked the time for a thorough member check with our participants, which is considered a limitation of this study. We did, however, use the tentative knowledge of life values from the first interviews and the literature exploration to check the results in later interviews. In this way the analysis is grounded and was performed by two experienced researchers.. Second, with the development of a generic value clarification tool for older adults with cancer as the final purpose of this research, the decision was made to focus on older people in general (> 70-…) instead of on more specific age groups. On the one hand, this decreased the chances for saturation with respect to older patients in general given the small sample seize, but on the other hand increased opportunities for the inclusion and still these are all patients in the last season of life (e.g., finished work life) However, even with a small sample size a lot of themes were discussed in all older age groups, still leading to partial saturation. For one of this article’s most interesting results, that older adults still have a strong wish to continue life although usually in a different style and mindset, the limited sampling in the group of the oldest old (*n* = 1 in this study) prohibits us from extrapolating the results to this age group as well. Finally, only HCPs with experience in palliative care were included, which might have biased the findings.

## Conclusion

This paper shows that older adults living with incurable cancer have their individual outlooks on life and life values that accompany the last phase of life. Healthcare professionals recognizing these outlooks and values, however, also express practical barriers in organizing shared explorations of such issues. By developing a value-clarification tool, patients as well as HCPs could be better supported in making well-considered and shared decisions for treatment and care.

## Supplementary information

**Additional file 1.** Interview guide – interviews with older adults living with incurable cancer.

**Additional file 2.** Topic guide focus groups Dutch Cancer Society-program ‘Older adults with cancer’.

## Data Availability

The anonymized dataset used and/or analyzed during the current study are available from the corresponding author on reasonable request.

## Abbreviation

HCPs: Healthcare professionals
