# Supplementary material for: Living and dying with incurable cancer: a qualitative study on older patients’ life values and healthcare professionals’ responsivity
Source: BMC Palliat Care. 2020 Jul 20;19:109. doi: 10.1186/s12904-020-00618-w (PMC7372747; doi:10.1186/s12904-020-00618-w)
Supplement: Supplementary file 1 — Additional file 1. Interview guide – interviews with older adults living with incurable cancer. [file 12904_2020_618_MOESM1_ESM.pdf]

Interview guide – interviews with older adults living with incurable cancer

* *Introduction*

- Personal introduction
- Aim of the study/aim of this particular interview
- Audio recording/ taking notes
- Time needed for this interview. The opportunity to stop this interview at any moment without having to provide an explanation.

Part 1: your experiences with care since you received your diagnosis

I would like to know how you experience the care you have received (use probing questions!)

*** Can you tell me a bit more about the illness that you live with?**
*** What do you expect to be the course of this illness?**
*** Which choices do you think you'll have to make in the nearby future?**
*** With whom are you discussing your illness? What then are the usual conversation topics?**

Now, I would like to focus more on your relationships with health care professionals. Let me first ask you a general question

*** How do you like to be treated by other people now that your are ill?**
* **Any ideas about how you would like to be treated the nearby future?**
Set up of a card game: participants provide answers in the form of attitudes or values, which in *a first step* are written down on cards.

*** Which health care professionals are involved after you have received the cancer diagnosis?**
In a *second step* of the card game the names/functions of the health care professionals are written down on a large piece of paper, and participants can 'play' with the value and

attitude cards to show which cards can be attributed to which HCPs. In the meantime, the researcher asks to explain these attributions: what do HCPs do/how do the act; which relationships are considered special and why;<br>The card game offers ample opportunities to look for contrast in the different relationships.
Summary
**Part 2: your experiences with your social environment**<br><br>Let's move on to our second conversation topic. I would like to know from you what your social life looks like these days.<br><br>*** With whom have you been in contact the last week** (probing on the how)?<br>*** the last month** (with an exception for the people already mentioned in response to the first question)<br>*The names and roles of these people are written down on cards*<br>*** perhaps there are people that you have missed these last days, weeks, months?** (probing on who, why, and what it is like)<br><br>Again we play the card game (to complete the network surrounding the patient):<br>*** What do you do with these people; what do they do with you?**<br>*** In what ways do they help you?**<br>*** What would you miss if they were not with you?**
Summary
**Part 3: hopes and preferences for the last phase of life**<br><br>*** How do you want to spend the time that's left?** (what do you want to do? What do you consider meaningful/worth doing?)<br>* What do you wish for?

* What do you expect to happen?<br>*** What is your preferred place of death? And why?**<br>*** Who is providing you with support?**<br><br>We have mainly discussed your social relations and your thoughts concerning your illness, including your hopes and desires. With your permission, I would like to ask some questions about the bodily aspects of your disease. Are you okay with that?<br>*** What are the physical consequences of being ill?**<br>*** What do you expect to happen to your body in the future?**<br><br>*** Did you ever think about whether or not wanting to go to the ICU**
Summary
**Part 4: self-perception**<br><br>I've got one final question for you:<br><br>*** If you look at yourself now and compare that with your old self (before you got ill) what are striking a)similarities and b) differences?**
Saying thanks and closing the interview
