# Supplementary material for: Living and dying with incurable cancer: a qualitative study on older patients’ life values and healthcare professionals’ responsivity
Source: BMC Palliat Care. 2020 Jul 20;19:109. doi: 10.1186/s12904-020-00618-w (PMC7372747; doi:10.1186/s12904-020-00618-w)
Supplement: Supplementary file 2 — Additional file 2. Topic guide focus groups Dutch Cancer Society-program ‘Older adults with cancer’. [file 12904_2020_618_MOESM2_ESM.pdf]

Topic guide focus groups Dutch Cancer Society-program 'Older adults with cancer'

- Word of welcome
- Introduction of the project
- Aim of the focus group
- Set up of the focus group

In the focus group, participants are invited to discuss with each other 5 vignette cases that contain all the main themes that arose from the patient study.

1. *Themes*: outlook an a 'reduced' life; suffering from pain, physical and/or mental deterioration, and depression; physician assisted suicide

Vignette synopsis: Mrs. M. (78 years old) lives with colon carcinoma and liver metastases. She has clear thoughts about what is no longer a life worth living. She seriously considers euthanasia, when the pain becomes to intense and she ends up laying in bed all day.

Questions

- Do you have similar cases in your daily practice taking care of older adults with cancer? Does Mrs. M's attitude somehow differ from the attitudes of younger patients (and how)?
- Do you consider yourself responsible for a) her ideas about the course of her disease, b) her thinking towards a physician assisted suicide? And why so?
- What would be your conversation with Mrs. M.?
- What supportive and/or obstructing factors will you most likely encounter when taking care of Mrs. M.?

2. *Themes*: hope (for a miracle; for an extension of life, but mostly a life dedicated to partaking instead of creating); having to accept that you had a long and happy life

Vignette synopsis: Mrs. M. (78 years old) still hopes for a miraculous cure. However, she thinks this cure to be highly unlikely. Therefore, she has scaled down her hope and prays for maintaining her current quality of life for as long as possible. Dying now would not be a problem, as she had a long and happy life, but she would love to still be around for a little while.

Questions

- Do you have similar cases of hope in your daily practice taking care of older adults with cancer? Does Mrs. M's hoping attitude somehow differ from the attitudes of younger patients (and how)?

- What is your responsibility when it comes to the hopes of your older patients with cancer?
- How do you think about (the norm of) having to accept death as the conclusion of a long and meaningful life?
- What would be your conversation with Mrs. M.?
- What are potential barriers and facilitators for a conversation about hope with people like Mrs. M.?

3. *Themes*: coping with an unpredictable disease, continuation of 'the normal life'

Vignette synopsis: Mrs. V. (83 years old) has pancreatic cancer. The diagnosis turned her world upside down. Although she does not deny her disease, she likes to live a 'normal life' and expects others to act normal as well.

Questions

- Do you have similar cases in your daily practice taking care of older adults with cancer? Does Mrs. V's attitude of 'acting normal' somehow differ from the attitudes of younger patients (and how)?
- Do you consider yourself responsible for how Mrs. V. rearranges her suddenly chaotic life due to the diagnosis? And why so?
- What would be your conversation with Mrs. V.?
- What supportive and/or obstructing factors will you most likely encounter when discussing this theme with Mrs. V.?

4. *Themes*: proxies, social relationships; being ill and being a family caregiver at the same time

Vignette synopsis: Mr. K. (86 years old) has been having prostate cancer for years now. Recently, bone metastases have surfaced. Just a few months earlier his wife was diagnosed with Alzheimers, leaving him with great worries about his wife's well being. He has opened up to his friends about his problems, and feels that their conversations led to more intense relationships. His children, living far away, are less involved.

Questions

- Do you have similar cases in your daily practice taking care of older adults with cancer?
- Do you consider yourself responsible for Mr. K's worries and existential questions?
- What supportive and/or obstructing factors will you most likely encounter when trying to discuss these themes with Mr. K.?

5. *Themes*: expectations from health care professionals; 'certainty' of technological medicine; not a single focus on palliative care; time and continuity of care

Vignette synopsis: The first time that Mr. S. (85 years old) hears about palliative care from his GP, he became suspicious. Wasn't that too soon? What about more extensive diagnosis, medical imaging? He hasn't seen the cancer in his intestines yet.

A few weeks later Mr. S. came home frustrated: he saw yet another specialist and had to do his story all over again. It left them with less time, leaving him with all kinds of questions after his meeting with the physician.

Questions

- Do you have similar cases in your daily practice taking care of older adults with cancer? Is this case characteristic for *older* adults with incurable cancer?
- What is your responsibility when it comes to Mr. S's uncertainty about diagnosis and medical imaging?
- What are potential barriers and facilitators for a conversation about these topics with people like Mr. S.?
